# E-cadherin on epithelial–mesenchymal transition in thyroid cancer

**DOI:** 10.1186/s12935-021-02344-6

**Published:** 2021-12-20

**Authors:** Xiaoyu Zhu, Xiaoping Wang, Yifei Gong, Junlin Deng

**Affiliations:** Shanghai Municipal Hospital of Traditional Chinese Medicine, Shanghai University of Traditional Chinese Medicine, 274 Zhijiang Middle Road, Jing’an District, Shanghai, 200040 China

**Keywords:** E-cadherin, Thyroid cancer, Review, Signal pathway

## Abstract

Thyroid carcinoma is a common malignant tumor of endocrine system and head and neck. Recurrence, metastasis and high malignant expression after routine treatment are serious clinical problems, so it is of great significance to explore its mechanism and find action targets. Epithelial–mesenchymal transition (EMT) is associated with tumor malignancy and invasion. One key change in tumour EMT is low expression of E-cadherin. Therefore, this article reviews the expression of E-cadherin in thyroid cancers (TC), discuss the potential mechanisms involved, and outline opportunities to exploit E-cadherin on regulating the occurrence of EMT as a critical factor in cancer therapeutics.

## Introduction

Thyroid cancer (TC) is one of the common head and neck tumors, which incidence rate increasing year by year. According to statistics, thyroid cancer is the fourth most common cancer in women, accounting for 5.7% of female malignant tumors [1]. The pathological classification included differentiated thyroid carcinoma (DTC) and undifferentiated thyroid carcinoma (UTC). Both of them can have lymph node metastasis, lung metastasis and are more likely to have distant and early bone metastasis, which seriously affect the prognosis and survival of patient [2]. Therefore, it is of great significance to explore the regulatory mechanism related to the metastasis of thyroid cancer. In the related studies of tumors, it has been shown that the malignant expression, metastasis and invasiveness of tumors are all related to epithelial–mesenchymal transition (EMT) [3].

Epithelial–mesenchymal transition (EMT) means that epithelial cells, stimulated by certain cancer-promoting factors, lose epithelial characteristics and their stable cell structure is destroyed and their cytoskeleton recombines, which leads to the weakening or disappearance of intercellular connections, eventually causing cells to express interstitial strong interstimic properties [4]. EMT in malignant tumor is a necessary process of metastasis and invasion, participating in the growth and development of cells and regulating the adhesion of cells [5–8]. The hallmarks of EMT are low expression of the epithelial marker E-cadherin, and high expression of the mesenchymal markers N-cadherin and Vimentin [4].

E-cadherin, also known as epithelial-cell adhesion molecule cadherin-1 (Cadherin1, Cdh1), is one of the classical adhesion proteins in the calcium adhesion superfamily, mainly expressed in epithelial cells [8, 9]. E-cadherin maintains cell adhesion and epithelial structural integrity, becoming a key marker for regulating the occurrence of EMT [5, 7, 10]. Based on its physiological characteristics, the increased invasiveness of epithelial tumors is related to the low expression of E-cadherin [8]. For example, in colorectal cancer, gastric cancer, breast cancer and other cancers, E-cadherin has been identified as a tumor suppressor gene affecting epithelial mesenchymal transformation, and is closely related to TNM stage, lymph node metastasis, extracapsular invasion and low disease-free survival rate [11, 12]. Moreover, in recent years, there are more and more studies on the inhibition of epithelial mesenchymal transformation and metastasis of thyroid cancer by regulating the expression of E-cadherin [13, 14]. It has been found that E-cadherin in TC is regulated by many signal pathways such as Wnt, PI3K/AKT, ERK1/2 and NF-κB. Therefore, the expression and regulation mechanism of E-cadherin in TC are reviewed signaling pathway of regulating E-cadherin, immune microenvironment, RNA and extracellular matrix to provide targets and new ideas for early diagnosis and treatment of thyroid tumors.

## Signaling pathway of regulating E-cadherin

### Wnt/β-catenin signaling pathway involved in the regulation of E-cadherin

Wnt signal pathway includes classical Wnt pathway (Wnt/β-catenin), non-classical Wnt pathway and Wnt/Ca2+ pathway, among which Wnt/β-catenin signal pathway is the most widely and fully studied pathway [15]. The activity of Wnt/β-catenin signaling pathway depends on the content of β-catenin in the cytoplasm [16]. When the Wnt signal is activated, the Wnt ligand binds to the receptor complex and then inhibits the phosphorylation of β-catenin, resulting in the accumulation of β-catenin in the cytoplasm and nuclear transfer to regulate the downstream target genes to affect the apoptosis and proliferation of the cells [16, 17].

The activation of Wnt/β-catenin signal pathway promotes the proliferation and stem cell characteristics of TC [15]. FOXN3, acting as anti-tumor effect in thyroid tumors, inhibit the occurrence of EMT by down-regulating the expression of β-catenin protein and up-regulating the expression of E-cadherin [18]. However, after activating the Wnt/β-catenin signaling pathway, invasion and migration as well as the occurrence of EMT are promoted, manifested by low expression of E-cadherin [19].

In addition to participating in gene regulation as an intermediary of Wnt signal pathway, β-catenin binds to E-cadherin in the absence of Wnt signal, and forms adhesion junctions (adherin junction, AJ) with α-catenin and γ-catenin connections, thus playing the role of adhesion and maintaining cell-to-cell stability [20, 21]. Abnormal expression of the complex formed by β-catenin and E-cadherin is closely related to the metastasis and recurrence of thyroid tumors [22]. A clinical study has shown that abnormal expression of β-catenin and deletion of E-cadherin on membrane in PTC patients, which may lead to low survival rate of patients [23]. However, when the above abnormal conditions are improved, the tumor grows slowly [23]. The use of Wnt inhibitor (DKK-1) can cause the accumulation of β-catenin in the cytoplasm and promote its formation of a complex with E-cadherin to maintain cell stability and inhibit the occurrence of EMT, thereby reducing cell migration and invasion [24]. On the other hand, the abnormal expression of them is related to the malignant degree of TC. Studies have shown that as the degree of differentiation increases E-cadherin is mainly expressed in the membrane and cytoplasm, while β-catenin is expressed in the cytoplasm, while E-cadherin and β-catenin almost disappear in poorly differentiated TC, which is related to the higher the TNM [25].

### PI3K/AKT and MAPK signaling pathway involved in the regulation of E-cadherin

PI3K/AKT is a common intracellular signal pathway. It has been found that PI3K/AKT signal pathway not only participates in cell proliferation and apoptosis, but also plays an important role in tumor growth, metastasis and tumor angiogenesis [26, 27]. It has been found that the activation of P13K/AKT signal pathway is related to the low expression of E-cadherin in thyroid carcinoma [28]. The PI3K/AKT signaling pathway in thyroid carcinoma is closely related to the high expression of oncogenes and the low expression of tumor suppressor genes. For example, the high expression of oncogenes HPIP, CUX2, miR-107 and miR-144-3p in thyroid carcinoma can activate PI3K/AKT signal pathway and down-regulate the expression of E-cadherin to induce the occurrence of EMT [29–32]. However, the low expression of tumor suppressor genes miR-146b and ING5 in thyroid carcinoma can not inhibit the expression of PI3K/AKT, which leads to the low expression of E-cadherin and induces the occurrence of EMT [33, 34].

From the above, it can be seen that there are many genes activating PI3K/AKT signal pathway in thyroid carcinoma. The mechanism of PI3K/AKT regulating E-cadherin can be summarized as follows. It has been found that activating AKT can destroy the adhesion junction structure (composed of E-cadherin, α-catenin, β-catenin and p130Cas proteins) between tumor cells, showing low expression of related proteins [35]. And the activation of AKT can directly induce the expression of Snail and then down-regulate the expression of E-cadherin to promote EMT [35]. The activation of AKT also exists in the thyroid carcinoma, and its activation can lead to the low expression of E-cadherin to induce EMT [36, 37]. The activation from gene to PI3K/AKT signal pathway seems to be closely related to PTEN and BRAF (V600E). PTEN and BRAF (V600E) are confirmed mutant genes in thyroid carcinoma, and they are closely related to the clinical manifestations of thyroid carcinoma malignancy [36, 38, 39]. PI3K molecules are divided into three categories, among which class I PI3K is closely related to carcinogenesis [40, 41]. The phosphorylation of PIP2 located on the inside of the lipid membrane promotes the phosphorylation of PIP3 and after AKT is recruited to the membrane, they bind to form p-AKT and finally activates the PI3K/AKT signal pathway [42].

PTEN, as a tumor suppressor, can reverse the phosphorylation of PIP3 and the recruitment of AKT to the inner membrane by terminating the signal transduction of PI3K/AKT pathway [26, 43, 44]. For example, inhibition of miR-146b in TC can promote the expression of PTEN, then inhibit AKT activation, and promote the expression of E-cadherin to inhibit EMT [45]. In addition, other genes such as F-box11, HPIP and DTX can regulate E-cadherin through the PI3K/AKT pathway, but the mechanism is still unclear [29, 46, 47]. In addition, some studies have found that the activation of PI3K/AKT signal pathway may be related to M2-like macrophages [48]. It has been found that when M2-like macrophages are co-cultured with thyroid cancer cells, the secretion of IGF-1 and IGF-2 by M2-like macrophages can activate PI3K/AKT signal pathway to reduce the expression of E-cadherin, thus induce the occurrence of EMT [48].

Mitogen-activated protein kinase (MAPK) pathway is a pathway that activates mitogen-activated protein (MAP) through a phosphate cascade signal cascade (Ras/RAF/MEK/ERK), via intracellular cascade proteins that eventually transfer signals into the nucleus to participate in process such as embryonic development, cell differentiation, proliferation and death [49, 50]. It has been found that RET and TRK rearrangement, BRAF and Ras mutations and B-Raf kinase in MAPKs signal pathway in TC lead to abnormal expression of MAPK/ERK, JNK/SAPK and p38/MAPK signal pathway [50, 51]. Silencing Kin17, DPP4 in TC can inhibit ERK1/2, JNK1 and P38, while promote E-cadherin, which may be related to the inhibition of MAPK/ERK, JNK/SAPK and p38/MAP signal pathways [52, 53]. MAPK/ERK signal pathway in TC can inhibit the expression of E-cadherin by promoting the expression of transcription factors Snail and slug [54].

### TGF-β involved in the regulation of E-cadherin

In tumors, TGF-β functions are complex and diverse due to different backgrounds and stages of tumors, not only acting as a suppressor to inhibit the occurrence and development of tumor cells in the early stage, but also to inhibit the invasion and metastasis in the late stage [55, 56]. TGF-β pathway includes classical and non-classical pathways, among which the classical TGF-β pathway plays a major role in the occurrence of EMT. Relevant literature has shown that at the later stage of tumor progression TGF-β affects the metastasis of tumor cells by down-regulating the expression of E-cadherin [57, 58]. For example, in thyroid follicular carcinoma membranous mice (ThrbPV/PV mice), the continuous activation of TGF-β/Smad2/3 signal pathway can reduce the expression of E-cadherin, thus promoting the occurrence of EMT [59, 60].

**Fig. 1:**
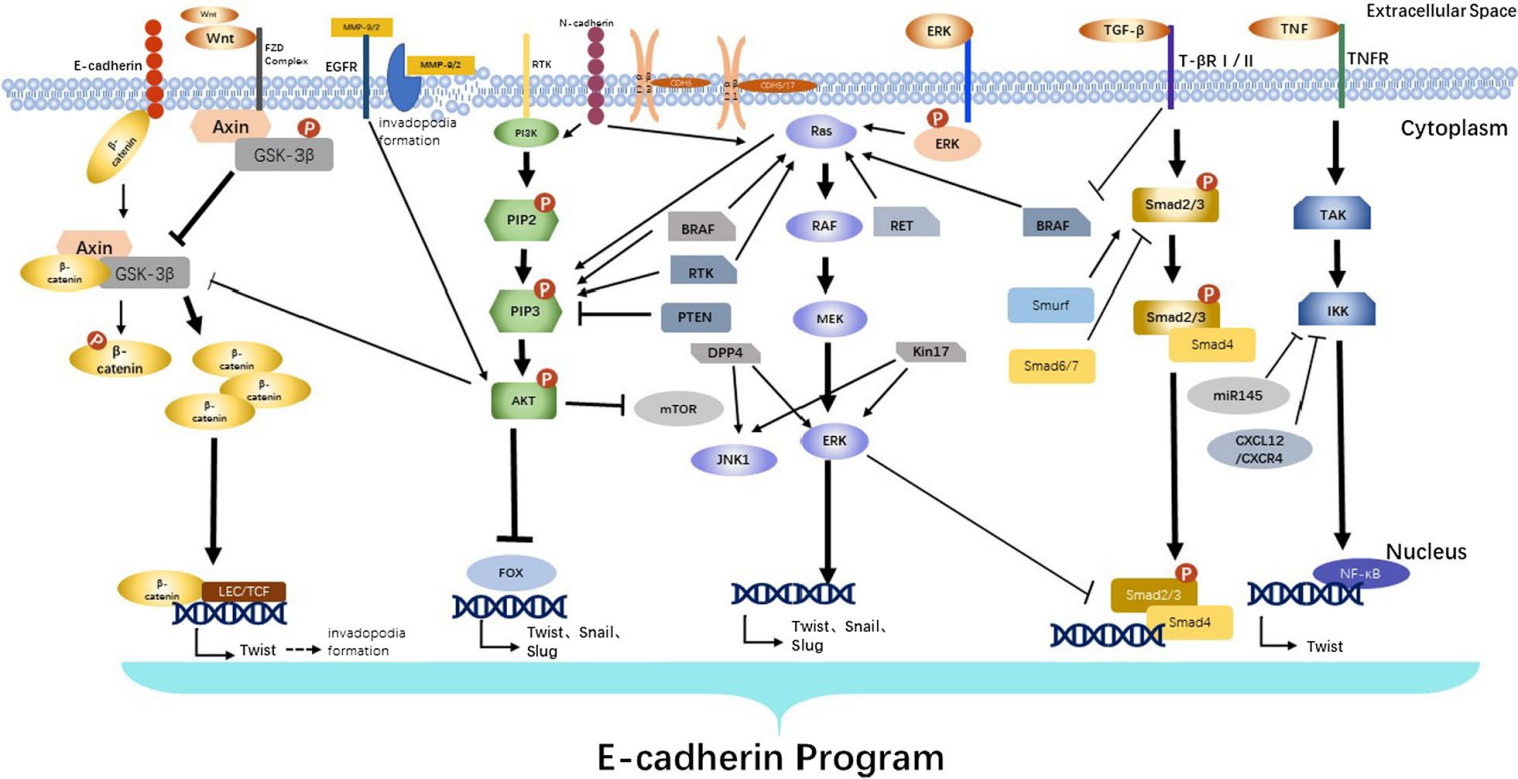
Pathway diagram for regulation of E-cadherin in thyroid cancer

The classical TGF-β signal pathway is called SMAD signal pathway, in which TGF-β ligand specifically binds to T-β RI and T β-RII receptors, transmits the signal into the cell, induces the phosphorylation of Smad2 and Smad3 and forms a complex with Smad4 to aggregate into the nucleus and regulate the expression of target genes, while Smad6 and Smad7 inhibit the generation of classical TGF-β signaling pathways by competitively binding T-βR to Smad2 and Smad3 [56]. It has been found that TGF-β regulates the expression of E-cadherin in TC through a variety of ways, thus participating in the invasion and metastasis of TC. For example, an experiment showed that by down-regulating Smurf, the ubiquitin ligase of Smad3, the TFG-β/SMAD pathway could be affected, thus down-regulating the expression of EMT marker E-cadherin in thyroid cancer [61]. Another study found that E-cadherin was inhibited by affecting the expression of TGF-β/Smad2/3 pathway [62, 63]. It may be related to the regulation of the transcription factor Snail and the tumor suppressor gene BRAFV600E. A study has shown that the BRAF^V600E^ gene mutation in thyroid tumors can down-regulate the expression of E-cadherin and induce the occurrence of EMT, and its abnormal expression in thyroid tumors increases the secretion of TGF-β, which further induces the occurrence of EMT and invasion, manifested as decreased expression of E-cadherin [64, 65]. However, the effect of TGF-β is extremely complex. Unlike the previous study, a clinical study found that the metastasis of thyroid tumors was not related to TGF-β but might be related to the overexpression of BRAFV600E in thyroid tumors and the activation of the transcription factor Snail, which decreased the expression of E-cadherin and ultimately promoted the occurrence of EMT [65]. This further indicates that TGF-β-related pathway can regulate the expression of E-cadherin and affect the occurrence of EMT, and thus affect the metastasis of thyroid cancer (Fig. 1).

## E-cadherin is related to the immunity/EMT/metastasis axis

The progression of malignant tumors is associated with inflammatory responses, and inflammation may be a key precipitating factor for the development of EMT during tumor progression [66, 67]. Tumor cells form a chronic inflammatory microenvironment by recruiting immune cells and inflammatory cytokines [68]. In the chronic inflammatory microenvironment created by tumor cells, some immune cells and soluble inflammatory mediators can recruit immunosuppressive cells, damage immune cells or reduce the immunogenicity of cancer cells, thus enabling abnormal cells to escape immune surveillance to form tumors [69]. Immune cells promote the diffusion and migration of tumor cells through matrix remodeling, induction of tumor cell invasion, migration and vascular infiltration [68]. This may be the mechanism of inflammatory reaction involved in tumorigenesis and development. Inflammatory cells continuously produce cytokines that induce EMT, while EMT can promote cancer cells to produce pro-inflammatory cytokines, which support each other and promote tumorigenesis and metastasis [70]. This form that EMT/inflammation axis. It can be seen that there is a bidirectional regulation between EMT and the immune system, and immune escape and immunosuppression play a key role in in tumor microenvironment. So this will be the focus of the next exposition.

### Immune cells involved in the regulation of E-cadherin

#### Innate immune cells involved in the regulation of E-cadherin

There are many kinds of innate immune cells in the immune microenvironment created by tumor tissues, in which macrophages and mast cells play an important role in the invasion and migration of thyroid cancer. As the largest number of immune cells in tumor microenvironment, macrophages can promote the malignant progression of tumor [71, 72]. Existing studies have shown that the polarization of macrophages into M2 macrophages (MA-TAMs) in tumor microenvironment is closely related to the malignant phenotype of tumor [70]. M2 macrophages can stimulate angiogenesis, inhibit T cell activity and release soluble cytokines to make tumor cells aggressive [73, 74]. A large number of studies have shown that M2 macrophages induce EMT by down-regulating the expression of E-cadherin [75–77]. In thyroid tumors, M2 macrophages can down-regulate the expression of Ecadherin, promote cell invasion and migration, and induce the occurrence of EMT [78]. There are a large number of inflammatory factors (such as TGF-β, TNF-α and IL) in the polarized environment of macrophages [76, 79]. These inflammatory factors can down-regulate the expression of β-catenin, thus inhibit the activation of Wnt/β-catenin signal pathway, and then up-regulate the expression of Ecadherin and inhibit EMT [77, 80]. In addition, the up-regulation of Wnt1 and Wnt3a is also present in the M2-TAM environment of thyroid cancer [78]. After gene knockout of Wnt1 and Wnt3a, the expression of β-catenin can be significantly decreased, then the Wnt signal pathway can be inhibited to up-regulate E-cadherin to inhibit EMT [78].

In addition, it was found that mast cells could also promote the occurrence of tumor EMT [81–83]. Mast cell infiltration also exists in the microenvironment of thyroid carcinoma, and the infiltration density of mast cells is closely related to the invasion of thyroid carcinoma [84]. Soluble factors (such as IL-8, IL-6, TBF- α, CXCR-1) and VEGF secreted by thyroid cancer cells can activate macrophages and recruit macrophages into tumor tissues [84, 85]. On the other hand, the activated macrophages secreted vesicles and mast cell chymase (MCC) could degrade the extracellular matrix and inhibit the expression of E-cadherin by overexpression of transcription factors Slug and Snail, thus inducing the occurrence of EMT [28, 86]. Or activated mast cells regulate the occurrence of EMT by regulating downstream kinases and promote tumor invasion and metastasis [85]. Thus a biphasic regulatory loop is produced in the local part of the tumor. A study shows that mast cells activated by thyroid cancer may induce EMT through IL-8-p-Akt-Slug signal pathway, and transcription factor Slug activation can inhibit the expression of E-cadherin and promote EMT [85]. To sum up, there is a biphasic regulatory loop between thyroid carcinoma and mast cells in the tumor. The cytokines produced by cancer cells activate mast cells, and mast cells promote the expression of transcription factors by activating downstream kinases, thus affecting the occurrence of EMT by regulating Ecadherin.

#### Adaptive immune cells involved in the regulation of E-cadherin

Many studies have shown that chronic inflammation has a pro-cancer effect in TC [87]. Immunosuppression of tumor infiltrating lymphocytes has been confirmed in advanced cancer. Similarly, this feature also exists in thyroid cancer. Through the immunohistochemical study of the tumor tissues of 74 patients with PTC, it was found that the characteristics of EMT (positive expression of E-cadherin, negative expression of Vimentin) and down-regulation of CD8+ surface markers occurred at the same time, and the characteristic changes were more significant in patients with lymph node metastasis and lymphatic invasion [88]. Through literature search, this process may be related to PD-1, a programmed death protein [89]. PD-1 exists on the surface of activated T cells, which mainly inhibits the activity of immune cells and regulates peripheral immune homeostasis [89]. Its ligand, programmed cell death ligand (PD-L1), as an immune checkpoint, its activation with EMT and its role in inhibiting tumor immune response have been confirmed [90–92]. There is also overexpression of PDL1 in thyroid carcinoma. It was found that there was a significant positive correlation between the positive expression rate of PD-L1 and mesenchymal phenotype in patients with PTC (P = 0.012), and the correlation between PD-L1 and EMT was enhanced in patients with recurrent and metastatic disease [88]. The induction of PD-L1 overexpression in 8505C and K1 cells could significantly promote the occurrence of EMT, showing the transformation of pebble-like cells into extensive spindle-like cells [88]. The above results show that the activation of EMT is related to PD-L1. This regulatory process, on the one hand, is due to the fact that the promoter region of PD-L1 gene contains Zeb1 binding sites [93, 94]. Zeb1 is a transcription factor of E-cadherin, which can induce EMT and maintain the stem cell nature of cancer cells to contribute to tumorigenesis [93]. A study found that Zeb1 can relieve the inhibition of PD-L1 in tumor cells, resulting in a decrease in the number of CD8+ T cells and an increase in the percentage of exhausted CD8+ T cells, leading to immunosuppression and metastatic nodules [95]. Another part of the view is that the occurrence of thyroid cancer EMT is related to glycosylation. A large number of studies have confirmed the key role of abnormal glycosylation in malignant tumors [96, 97]. Similarly, there is abnormal glycosylation in thyroid carcinoma, which may be related to FTU8, a fucosyltransferase [98]. FTU8 can mediate core fucosylation and thus participate in tumor invasion and metastasis [99, 100]. Through the study of lung and colon cancer, it was found that the up-regulation of FUT8 could increase the core fucosylation level of E-cadherin and decrease the expression of Ecadherin protein [99, 101]. There are N-glycosylation sites on the surface of PD-1 protein on T cell surface [102]. Through FTU8 gene knockout or drug inhibition of FUT8 activity, it can inhibit the expression of PD-1 on T cell surface, thus promote the activation of T cells and inhibit tumorigenesis [102]. Through the preparation of FUT8 gene knockout and FUT8−/− mouse model, it was found that the activity of FUT8 regulates the total amount of E-cadherin [99]. Therefore, the regulation of EMT by T cells through E-cadherin may be related to PD-1, and this regulation process may be related to transcription factor Zeb1 and glycosylation.

### Inflammatory factors involved in the regulation of E-cadherin

Inflammatory cytokines play an important role in maintaining inflammatory microenvironment and tumor progression [103]. Abnormal expression of different types of cytokines can be found in different types of cancer. For example, in breast cancer [104], lung cancer [105] and liver cancer [106], the abnormal expression of inflammatory cytokines is closely related to the progression of tumor migration. There is also abnormal expression of inflammatory cytokines in thyroid carcinoma. The expression of IL-34 was up-regulated in serum and tissue samples of PTC, and the overexpression of IL-34 was significantly correlated with distant metastasis and lymphatic metastasis of tumor [13, 107]. Tumor necrosis factor-α (TNF-α) and interferon-γ (IFN-γ) can induce the malignant development of thyroid cancer cell line, change the cell morphology from fat circle to spindle shape gradually, increase the invasion and migration ability of cells, decrease E-cadherin and increase the expression of N-cadherin and Vimentin at protein level [101, 108]. After stimulated by TNF-α and IFN-γ, E-cadherin was located in the cytoplasm and N-cadherin was located on the cell membrane, and the cancer cells showed interstitial characteristics [101]. A study found that IL-1a can cause ultrastructural changes in thyroid follicular cells, reduce the tightness between thyroid epithelial cells, and lead to the disintegration of intercellular adhesion junction structure (AJ), composed of E-cadherin and α-catenin, β-catenin, γ-catenin [109]. Further study found that the decreased expression of E-cadherin protein in thyroid cancer cell lines stimulated by inflammatory factors may be related to the activation of transcription factors Snail, Slug, Twist1 and Zeb1 [14]. This regulatory process can inhibit the invasion and migration of thyroid cancer cells induced by inflammatory cytokines by using AKT, NF-κB and SATAT3 signaling pathway [14]. Nuclear factor kappa B (NF-κB) is a transcription factor that regulates apoptosis, inflammation and immune response [110]. With studies suggesting that NF-κB may be a key link between inflammation and cancer, it’s overactivation is related to the proliferation, angiogenesis and metastasis of many malignant tumors and can serve as a potent inductor of EMT [110–112]. Similarly, the abnormal activation of NF-κB in thyroid carcinoma has been shown to be related to tumor proliferation, invasion and migration [113, 114]. Through the study of NF-κB in other tumors, its regulatory mechanism may be related to the NK-b/Twist axis [115, 116]. After knockout of NF-κB, the proliferation and migration ability of cells stimulated by TNF-α decreased, and the expression of E-cadherin was up-regulated, N-cadherin and Twsit was down-regulated [115]. However, the inhibitory effect of knockout Twist was the same as before [115]. After the thyroid cancer cells were treated with TNF-α, the expression of Twist mRNA was up-regulated and then the expression of E-cadherin was down-regulated [116]. After the use of NF-κB inhibitor, the expression of Twist mRNA and protein was inhibited, and the expression of E-cadherin protein was up-regulated [116]. To sum up, inflammatory cytokines may activate transcription factors through signal pathways, thus down-regulating E-cadherin from promoting the occurrence of EMT.

## E-cadherin is related to RNAs

### MicroRNAsinvolved in the regulation of E-cadherin

MicroRNAs (miRNAs or miRs) are non-coding small RNA that is transcribed from DNA into a precursor miRNAs (pri-miRNAs) in the nucleus and processed into mature miRNAs for export to the cytoplasm, where they can then degrade or regulate mRNA by targeting the 3′-untranslated region, thereby regulating cell growth, cycle, apoptosis and other biological processes [52, 117]. MiRNA can be used as both a tumor suppressor gene and a tumor promoting gene to regulate the occurrence of tumor EMT in thyroid carcinoma. For example, overexpression of miR-203, MIR-597-3p, MIR-200b, miR-122 and miR-146b-5p significantly increase the expression of E-cadherin and decrease the expression of Ncadherin or Vimentin [118–122]. On the contrary, MiR-144-3p and miR-483-3p in thyroid carcinom, as an oncogene, is overexpressed in PTC to reduce E-cadherin to induce EMT [123, 124].

Although miRNAs is abundant in thyroid carcinoma, its expression is high or low to regulate the expression of Ecadherin. The regulatory mechanism is that miRNA affects target gene transcription by binding to the 3′-UTR of target gene mRNA [52, 117]. For example, in TPC-1 cells, miR-599 as a suppressor gene, up-regulation of its expression can be targeted to bind to the 3'-UTR of Hey2, thus down-regulating its expression [125]. Down-regulation of Hey2 expression inhibits Notch signal pathway, suppresses the expression of transcription factors Snail and Slug, thus up-regulates the expression of E-cadherin and inhibits the occurrence of EMT [125]. MiR-483-3p inhibits the expression of PARD3 by targeting the 3′-UTR of it, and then up-regulates the expression of Snail, Slug, Zeb1 and Twist to inhibit the expression of E-cadherin and finally induce EMT [124]. MiR-31 upregulates the expression of E-cadherin through targeted inhibition of Sox11 to inhibit ERK and AKT signaling pathways, thus inhibiting EMT [126]. In addition, miR199a-5p can directly inhibit the expression of Snail to up-regulate the expression of E-cadherin and induce EMT [127]. Many other situations in which miRNAS regulates E-cadherin are shown in Table 1.

**Table 1:** The regulatory mechanism of miRNAs on E-cadherin in thyroid cancer cells

miRNAs	Status in TCs	Cell line origin	Regulation and control approach	Evidence of regulating E-cadherin in TCs	References
miR-203	L	TPC-1	Up-regulation of AKT	Low expression of E-cadherin Induction of EMT T	[118]
miR-597-3P	L	SW579	Targeted up-regulation of RAB23	Low expression of E-cadherin Induction of EMT	[119]
miR-599	L	TPC-1	Targeted inhibition of Hey2 to inhibit Notch signaling pathway	High expression of Snail and Slug, low expression of E-cadherin Induction of EMT	[125]
miR-20b	L	K1, TPC-1	Up-regulating SOS1 and ERK2 to activate MAPK/ERK signaling pathway (up-regulation of p-MEK1/2, p-ERK1/2, t-ERK2)	Low expression of E-cadherin Induction of EMT	[128]
miR-199a-5p	L	8505C, SW1736	Targeted up-regulation of Snail	Low expression of E-cadherin Induction of EMT	[127]
miR-199a-5p	L	b-CPAP, SW579	Targeted up-regulation of STON2	Low expression of E-cadherin Induction of EMT	[129]
miR-215	L	K1, b-CPAP, TPC-1, IHH4	Up-regulating ARFGEF1 to activate AKT/GSK-3β signaling pathway (up-regulation of p-AKT, p-GSK-3β)	High expression of Snail, low expression of E-cadherin Induction of EMT	[130]
miR-451a	L	b-CPAP, KTC-1	Targeted up-regulation of PSMB8	Low expression of E-cadherin Induction of EMT	[131]
miR-613	L	K1, TPC-1, b-CPAP	Targeted up-regulation of TAGLN2	Low expression of E-cadherin Induction of EMT	[132]
miR-630	L	SW1763, TPC-1	Activation of JAK/STAT3 signal path (up-regulation of p-JAK、p-ATAT3)	Low expression of E-cadherin Induction of EMT	[133]
miR-483-3p	H	8580C, FRO	Targeted inhibition of PARDS	High expression of Snail, Slug, Zeb1 and Twist, low expression of E-cadherin Induction of EMT	[124]
miR-31	L	TPC-1, b-CPAP	Up-regulating Sox11 to activate ERK and AKT signaling pathway (up-regulation of p-ERK1/2, p-AKT)	Low expression of E-cadherin Induction of EMT	[126]

*TCs* thyroid cancer cells, *H* high expression, *L* low expression

### lncRNAs involved in the regulation of E-cadherin

LncRNAs are a type of coding gene that transcribes > 200 nucleotides in length and don’t encode protein, acting as a separate transcriptional unit, an intron of an enhancer (ERNAs) or promoter [134]. Cancer is now thought to occur in association with genetic mutations, and since the coding genome accounts for less than 2% of all sequences, studies suggest that cancer may also be driven by aberrations in the non-coding genome [53]. lncRNAs participate in the regulation of tumor cell proliferation, growth inhibition, invasion and metastasis and EMT [53, 135]. In thyroid tumors, the regulation of EMT by lncRNAs is bidirectional. The overexpression of lncRNAs may lead to the low expression of E-cadherin in thyroid tumors and cancer cells, which can induce EMT, while the low expression of lncRNA may also occur the above process. For example, there are high expressions of lncRNA-HOTAIR, lncRNA-NORAD, lncRNA-linc00673, lncRNA-SLC26A4-AS1, lncRNA-ROR, lncRNA-PVT1, lncRNA-HOTAIR and other lncRNAs in thyroid cancer patients, which are positively correlated with tumor malignant phenotype and lymph node metastasis, while lncRNAs knockout in thyroid cancer cells can up-regulate the expression of E-cadherin protein and inhibit the occurrence of EMT [19, 136–140]. On the contrary, the low expression of lncRNA-CASC2 and lncRNA-linc 00106 in thyroid carcinoma was positively correlated with lymph node metastasis, and up-regulation of lncRNAs could up-regulate the expression of E-cadherin protein and inhibit the occurrence of EMT [141–143]. In addition, the expression level of the same lncRNA is also different in different thyroid cancer tumors. For example, the expression level of lncRNA-BANCR in bCPAP is lower than that of CAL-62, WRO and FTC-133 [144]. MALAT1 is highly expressed in PTC, as an oncogene to promote tumor EMT, and as a tumor suppressor gene to inhibit tumorigenesis in poorly differentiated and anaplastic thyroid carcinoma [145].

There are many kinds of lncRNAs, but the mechanism of regulating E-cadherin can be summarized as the following three points. First of all, lncRNAs can play a sponge role on miRNAs to inhibit the expression of miRNAs, thus affecting the expression of downstream transcription factors by regulating related signal pathways to regulate the expression of E-cadherin, and finally regulate the occurrence of EMT. Overexpression of LncRNA-UCA1 can down-regulate the expression of transcription factor Snail by activating Hippo and JNK signal pathways, and up-regulate the expression of E-cadherin to inhibit the occurrence of EMT [146]. In addition, lnc-TUG1, lnc-NORAD and LINC02471 inhibit the expression of E-cadherin by regulating the expression of miRNAs and promoting the expression of transcription factors Snail, Slug and Zeb1, thus promoting the occurrence of EMT [136, 147, 148]. Secondly, lncRNAs can directly regulate the expression of tumor-related genes, and then regulate the expression of E-cadherin. For example, KLF-2-like factor 2 (KLLF2), as a tumor suppressor gene, has low expression in thyroid carcinoma and is associated with lymph node metastasis, malignant histological type and high TNM stage [149]. LIN00673 is highly expressed in thyroid carcinoma and knockout of LIN00673 can enhance the expression of KLF-2 and increase the expression of E-cadherin, thus inhibit the occurrence of EMT [149]. On the contrary, CRABP2 (retinoic acid binding protein 2) is a necessary protein for tumor growth [150]. Overexpression of LINC01816 in thyroid carcinoma can target miR-34c-5p to act as a sponge, and then up-regulate the expression of CRABP2 and inhibit the expression of E-cadherin, thus promoting the occurrence of EMT [151]. Thirdly, LncRNAs can directly regulate the relevant signal pathways to regulate E-cadherin. The overexpression of lnc-BANCR in bCPAP cells can down-regulate the expression of E-cadherin and inhibit EMT by activating Raf/MEK/ERK signal pathway [144]. LncRNA-HOTAIR is highly expressed in thyroid carcinoma [19]. Inhibition of HOTAIR can decrease the expression of Wnt inhibitory factor (WIFI), up-regulate β-catenin, activate Wnt/β-catenin signal pathway, and then down-regulate E-cadherin to promote the occurrence of EMT [19]. On the contrary, knockout of LINC00106 gene can down-regulate E-cadherin and β-catenin, and induce the occurrence of EMT [152]. LINC00313 promotes the methylation of ALX4 promoter by recruiting methylated proteins, while LINC00313 knockout can inhibit the methylation of ALX4 [153]. The accumulation of ALX4 in the cytoplasm can inactivate the AKT/mTOR signal pathway and down-regulate the expression of E-cadherin, thus promoting the occurrence of EMT [153]. In thyroid carcinoma, lncRNA-ZFAS1 and its transcription factor GREB3 are up-regulated [154]. GREB3 activates the expression of lncRNA-ZFAS1 and then targets to inhibit the expression of miR373-3p/MMP-3 to up-regulation of Slug and Snail, and then down-regulation of E-cadherin to promote the occurrence of EMT [154] (Table 2).

**Table 2:** The regulatory mechanism of lncRNAs on E-cadherin in thyroid cancer cells

LncRNAs	Status in TCs	Cell line origin	Regulation and control approach	Evidence of regulating E-cadherin in TCs	References
UCA1	H	TPC-1	Down-regulation of miR-15a to activate Hippo and JNK signaling pathways (up-regulation of p/t-MST, p/tYAP, p/t-c-jun和p/t-JNK)	High expression of Snail and Zeb1, low expression of E-cadherin Induction of EMT	[146]
TUG1	H	FTC-133	Down-regulation of miR-145	High expression of Zeb1, low expression of E-cadherin Induction of EMT	[147]
NORAD	H	K1, bCPAP, TPC1, NPA187	Down-regulation of miR-202-5p	High expression of Zeb1, low expression of E-cadherin Induction of EMT	[136]
LINC02471	H	TPC-1, IHH4	Down-regulation of miR-375	High expression of Snail, low expression of E-cadherin Induction of EMT	[148]
PAR5	L	8505C, FRO	Up-regulation of EZH2	Low expression of E-cadherin Induction of EMT	[143]
ZFAS1	H	TPC-1	Inhibition of miR-373-3p/MMP-3 axis	High expression of Snail and Slug, low expression of E-cadherin Induction of EMT	[154]
LINC00637	H	TPC-1, bCPAP	Down-regulation of Kruppel-like factor 2(KLF2)	Low expression of E-cadherin Induction of EMT	[149]
LINC01816	H	C643	Sponge action on miR-34c-5p to upregulate CRABP2	Low expression of E-cadherin Induction of EMT	[151]
BANCR	H	bCPAP	Activation of Raf/MEK/ERK (up-regulation of p-c-Raf, p-ERK1/2, p-MEK1/2)	Low expression of E-cadherin Induction of EMT	[144]
LINC00106	L	bCPC, TPC-1	Down-regulation of β-catenin	Low expression of E-cadherin Induction of EMT	[152]
HOTAIR	H	TPC-1	Activation of Wnt/β-catenin (down-regulation of WIFI, up-regulation of β-catenin)	Low expression of E-cadherin Induction of EMT	[19]
LINC00313	H	TPC-1, SW579	The methylated proteins (DNMT1 and DNMT3b) promoted the methylation of ALX4 to activate AKT-mTOR signaling pathway (up-regulation of p-mTOR and p-AKT)	Low expression of E-cadherin Induction of EMT	[153]
N384546	H	KTC-1, bCPAP	Activation of miR-145-5p/AKT (down-regulation of miR-145-5p to activate Akt)	Low expression of E-cadherin Induction of EMT	[140]
SLC26A4-AS1	L	TPC-1	Activation of MAPK signal pathway (up-regulation of p-JNK1/2 and ERK, down-regulation of TP53)	Low expression of E-cadherin Induction of EMT	[138]

*TCs* thyroid cancer cells, *H* high expression, *L* low expression

## E-cadherin is related to extracellular matrix

### Matrix metalloproteinases family (MMPs) involved in the regulation of E-cadherin

Extracellular matrix participates in cell movement and apoptosis and provides cytoskeleton support, of which matrix metalloproteinases is the major components [155]. MMPs are zinc-dependent endopeptidases that degrade various protein components of the extracellular matrix (ECM) and are closely related to tumor invasion, metastasis, and angiogenesis [156–158]. In the early growth phase of tumor, MMPs inhibitor restrain tumor growth and degrade it [159]. MMPs promote the development of EMT in tumor cells by regulating a variety of regulatory factors [157]. TGF-β can promote EMT in TC, manifested as low expression of E-cadherin, while inhibiting the expression of MMP-9 can inhibit the occurrence [160]. EGFR is related to angiogenesis and tumor growth. MMP-2/9 activates EGFR to regulate E-cadherin through ERK1/2 and AKT/GSK3-β/β-catenin [161]. Twist (an E-cadherin transcription factor) can induce the formation of actin enrichment membrane protrusions that recruit MMP-7, MMP-9 and MMP-14 on the membrane to degrade locally and destroy the cytoskeleton [162]. The above studies may be a powerful explanation for the regulation of E-cadherin by MMPs to promote tumor invasion and migration.

### Different cadherins involved in the regulation of E-cadherin

Cadherin is a Ca2+-dependent intercellular adhesion molecule, which acts as a bridge between cells in the extracellular matrix and participates in the regulation of tumor invasion, migration and angiogenesis [163]. E-cadherin (CdH1), as a tumor suppressor of EMT, has a significant correlation with malignant phenotype, invasion and migration of tumor cells [164]. In addition, cadherin also includes CDH5 (VE-Cadherin), cadherin 6 (CDH6), cadherin 17 (CDH17), N-cadherin and so on, which play a similar role to E-cadherin in EMT, or regulate E-cadherin through some pathways.

Abnormal expression of N-cadherin in thyroid tumors significantly inhibits expression of E-cadherin, the conversion of E-cadherin to N-cadherin is the key factor to promote the development of malignant tumors [6]. A study has shown that E-cadherin expression can be up-regulated by inhibiting the expression of N-cadherin in thyroid tumors, possibly by promoting the expression of transcription factors Twist, Snail, and Slug to promote the occurrence of EMT and invasive migration [165]. Studies have shown that CDH16 appears to be another marker of EMT, regardless of E-cadherin negative or positive expression, CDH16 is negative and declined to a greater extent than E-cadherin [166]. RGD motifs in CDH17 and CDH5 promote tumor migration and invasion by activating α2β1 integrin signal and CDH6 activating αIIbβ3 integrin signal [167, 168]. Placental adhesin (CDH3) promote the growth, migration and invasion of TC, which may play a role by up-regulating E-cadherin and down-regulating N-cadherin [169]. E-cadherin is not expressed in TPC1 and bCPAP, but N-cadherin is down-regulated by silencing CDH6 to inhibit EMT, which may be regulated by the inhibition of forming autophagosome by the combination of LC3 and Flag-GABARAP after CDH6 silencing [170].

## Conclusion

The article reviews the expression of E-cadherin in TC and the related pathways regulating its expression. To sum up, it can be concluded that the low expression of E-cadherin in thyroid carcinoma promotes the invasion and migration of tumor cells, thus inducing the occurrence of EMT. The signaling pathways regulating this process include Wnt/β-catenin, PI3K/AKT, MAPK and TGF-β signaling pathways, as well as tumor microenvironment regulated by immune system, miRNA and lncRNA regulating oncogenes or tumor suppressor genes and extracellular matrix. By inhibiting E-cadherin, it reduces the contact between cells, induces the occurrence of EMT, and leads to the movement of thyroid tumor. From in vitro and in vivo studies, E-cadherin may be a promising biomarker for malignant phenotypes such as tumor invasiveness, distant metastasis and lymph node metastasis, which may make E-cadherin an indicator of early diagnosis and prognosis of TCs.

As the adhesion between protein-dimensional cells on the membrane, E-cadherin surface molecules show dysfunction when tumor cells metastasize and decompose into molecules soluble in extracellular matrix and blood, so the changes of E-cadherin can be detected in patient’s serum [171, 172]. Serum E-cadherin is higher in thyroid papillary carcinoma than in benign nodules and normal tissues, while the expression of E-cadherin in thyroid papillary carcinoma was lower than that in paracancerous tissues, and it was significantly correlated with malignant phenotypes such as lymphatic metastasis and distant metastasis [158, 172]. And the low expression of E-cadherin in cancer tissues may be related to the activation of its promoter methylation [13]. It has been found that thyroid cancers with lymphatic metastasis are more likely to have Ecadherin methylation and low expression of E-cadherin [13]. Thyroid tumors are divided into benign tumor, differentiated thyroid carcinoma, poorly differentiated thyroid carcinoma and undifferentiated thyroid carcinoma according to the pathological types [62]. With the increase of malignant degree, the positive expression rate of E-cadherin decreases gradually, which is negatively correlated with higher clinical stage, distant lymph node metastasis, extracapsular infiltration and lower disease-free survival rate [62, 173]. And in gastric cancer, breast cancer, ovarian cancer and other tumors also showed that the low expression of E-cadherin-induced EMT is related to the invasive characteristics of tumors, and become an independent prognostic factor of tumor patients [75]. Interestingly, in one study, there was no difference in the expression of E-cadherin among neoplastic, adenoma and non-neoplastic lesions [174]. We think this may be the result of the small sample size.

To sum up, it can be seen that the occurrence of malignant expression type of thyroid carcinoma is significantly related to the low expression of E-cadherin, whether in vivo or in vitro. This suggests that E-cadherin may provide a basis for the diagnosis of thyroid tumors and assist in malignant degree and pathological classification. By reviewing the mechanism of regulating E-cadherin, E-cadherin may provide a new idea for the treatment of thyroid cancer, which provides a wealth of options for treatment of TC, not limited to traditional surgery, radioiodine therapy and radiochemotherapy [175]. From E-cadherin to explore the treatment strategy of thyroid cancer include the following three aspects: (1) block the transduction of signal pathways related to the regulation of E-cadherin in thyroid carcinoma; (2) inhibition of expression of transcription factors upstream of E-cadherin; (3) immune activation regulates the tumor microenvironment of thyroid cancer.

The expression of E-cadherin is regulated by pathway inhibitors in thyroid carcinoma, which is mainly concentrated in Wnt/β-catenin, PI3K/AKT and MAPK signal pathways. The Wnt pathway inhibitor (DKK-1) reverses the deletion of E-cadherin expression in PTC cell membrane, thereby inducing EMT [176, 177]. High expression of β-catenin and abnormal localization of nucleus and cytoplasm were found in thyroid carcinoma [178]. In one study, the non-steroidal anti-inflammatory drug, sulinda hypothesis, decreased the expression of β-catenin in thyroid cancer cells and showed nuclear transfer to the cell membrane [178]. In addition, it was also found that the process of reversing β-catenin was only found in 8505murine C and KTC-1 cells with BRAF (V600E) mutation [178]. β-Catenin inhibitors can obviously inhibit the resistance of thyroid cancer cells to BRAF (V600E) inhibitors, thus inhibiting the occurrence of EMT [179]. Inhibition of EMT by inhibiting RTK and Akt/mTOR signaling pathways and promoting the expression of E-cadherin in thyroid cancer cells [180]. In clinical studies, kinase inhibitors Sorafenib, Vandetanib, Cabozantinib and Lenvatinib have been approved by FDA and the European Medical Association (EMA) for the treatment of medullary thyroid carcinoma and advanced RAI-R (refractory to radioiodine therapy) and poorly differentiated thyroid cancer [181]. A phase I clinical study found that thisirolimus, a mTOR inhibitor, enhanced the limited rate of sorafenib in the treatment of recurrent or metastatic radioiodine refractory thyroid cancer (RAITC), compared with sorafenib alone [182]. A retrospective study of clinical mortality and causes of death in patients with thyroid cancer found that the mortality rate of patients using tyrosine kinase inhibitor (TKI) (15.8%) was lower than that of patients undergoing surgery (64.2%), radiotherapy (53.3%) and cytotoxic chemotherapy (24.2%) [183]. Surgery and radiotherapy combined with TKI was the most effective, and the concomitant median survival rate was 34.3 months [183]. Distant metastasis has become the main cause of thyroid surface death in patients without TKI treatment [183].

A large number of studies have shown that malignant phenotype can be improved by regulating the expression of upstream transcription factors of E-cadherin. As an oncogene of thyroid cancer, BRAFV600E mutation can induce the development of thyroid cancer [39]. The carcinogenicity of BRAFV600E is caused by the activation of MAPK signal pathway [184]. The study found that thyroid cancer patients’ with BRAFV600E mutation were treated with BRAFV600E inhibitor (Dabrafenib) combined with MEK inhibitor (Trametinib) symptoms were relieved and the median survival time was increased [185, 186]. In addition, the efficacy of Dabrafenib combined with Trametinib in metastatic non-small cell lung cancer (NSCLC) and advanced melanoma with BRAFV600E mutation has also been confirmed, which can prolong the median progression-free survival (PFS) and global survival (OS) of tumor patients with less physical toxicity [187, 188]. In addition, there is the regulation of tumor suppressor factor PTEN. Resveratrol can promote PTEN expression and nuclear metastasis in thyroid cancer cells, and at the same time induce E-cadherin transfer from cytoplasm to cell membrane to enhance intercellular adhesion and inhibit the occurrence of EMT [189]. Green tea extract (EGCG) and Combretastatin A4 can directly inhibit transcription factors Snail, Slug, Zeb1 and Twist1 to up-regulate the expression of E-cadherin and inhibit the occurrence of EMT [190, 191].

It can be seen from the above that immunosuppression and immune escape are the main causes of immune system EMT in thyroid cancer. Then the corresponding immunotherapy includes immune checkpoint inhibitors and immunization. Immunosuppressive molecule PD-1 is overexpressed on activated T cell membrane and inhibits T cell function by binding to its ligand [89]. Therefore, the specific recognition of T cells to tumor immune antigens can be improved by immune checkpoint inhibitors in patients with T cell inflammatory phenotype [192, 193]. The therapeutic value of anti-PD-1 and PD-L1 drugs, pembrolizumab, nivolumab and atezolizumab, in recurrent or metastatic head and neck squamous cell carcinoma, non-small cell lung cancer and melanoma has been confirmed, showing that the patients have higher survival rate and safety [194–196]. And a meta analysis confirmed that anti-PD-1 drugs (nivolumab, pembrolizumab, atezolizumab) did not cause serious organ-specific immune adverse events such as pneumonia, colitis and hypophysitis, on the contrary, tumor-targeted drugs and chemotherapeutic drugs had a higher risk of organ-specific immune adverse events, with a high security [197]. The concentration of macrophages in tumor microenvironment was positively correlated with extraglandular invasion and extracapsular invasion, and negatively correlated with survival rate [198]. Therefore, inhibiting the polarization of macrophages may be a therapeutic idea. It has been found that zoledronic acid (ZA) can inhibit M2-like polarization of macrophages and inhibit the characteristics of thyroid cancer stem cells and the occurrence of EMT, which is characterized by low expression of stem cell markers CD133 and Oct4 and high expression of E-cadherin [199].

It can be seen from the above that in thyroid carcinoma, the expression of E-cadherin is affected by regulating signal pathways, upstream genes and immune microenvironment, thus affecting the occurrence of EMT. There are a variety of methods, whether they have been clinically proven, or are still in the experimental stage, or are simply studied in vitro. These results suggest that it is a new idea to regulate E-cadherin to affect the occurrence of thyroid EMT and improve the malignant expression of tumors.

Overall, the malignant phenotype of thyroid cancer is negatively correlated with E-cadherin, and its complex regulatory mechanisms and widely involved cytokines may provide new ideas for the early diagnosis, prognosis and treatment of thyroid cancer.

## Data Availability

Yes.
